# Next generation sequencing to dissect the genetic architecture of *KNG1* and *F11* loci using factor XI levels as an intermediate phenotype of thrombosis

**DOI:** 10.1371/journal.pone.0176301

**Published:** 2017-04-26

**Authors:** Laura Martin-Fernandez, Giovana Gavidia-Bovadilla, Irene Corrales, Helena Brunel, Lorena Ramírez, Sonia López, Juan Carlos Souto, Francisco Vidal, José Manuel Soria

**Affiliations:** 1 Unit of Genomics of Complex Diseases, Biomedical Research Institute Sant Pau (IIB-Sant Pau), Barcelona, Spain; 2 Department of ESAII, Center for Biomedical Engineering Research (CREB), Universitat Politècnica de Catalunya, Barcelona, Spain; 3 Congenital Coagulopathies, Blood and Tissue Bank, Barcelona, Spain; 4 Molecular Diagnosis and Therapy, Vall d'Hebron Research Institute, Universitat Autònoma de Barcelona (VHIR-UAB), Barcelona, Spain; 5 Unit of Hemostasis and Thrombosis, Department of Hematology, IIB-Sant Pau, Hospital de la Santa Creu i Sant Pau, Barcelona, Spain; 6 CIBER de Enfermedades Cardiovasculares, Barcelona, Spain; Institut d'Investigacions Biomediques de Barcelona, SPAIN

## Abstract

Venous thromboembolism is a complex disease with a high heritability. There are significant associations among Factor XI (FXI) levels and SNPs in the *KNG1* and *F11* loci. Our aim was to identify the genetic variation of *KNG1* and *F11* that might account for the variability of FXI levels. The *KNG1* and *F11* loci were sequenced completely in 110 unrelated individuals from the GAIT-2 (Genetic Analysis of Idiopathic Thrombophilia 2) Project using Next Generation Sequencing on an Illumina MiSeq. The GAIT-2 Project is a study of 935 individuals in 35 extended Spanish families selected through a proband with idiopathic thrombophilia. Among the 110 individuals, a subset of 40 individuals was chosen as a discovery sample for identifying variants. A total of 762 genetic variants were detected. Several significant associations were established among common variants and low-frequency variants sets in *KNG1* and *F11* with FXI levels using the PLINK and SKAT packages. Among these associations, those of rs710446 and five low-frequency variant sets in *KNG1* with FXI level variation were significant after multiple testing correction and permutation. Also, two putative pathogenic mutations related to high and low FXI levels were identified by data filtering and *in silico* predictions. This study of *KNG1* and *F11* loci should help to understand the connection between genotypic variation and variation in FXI levels. The functional genetic variants should be useful as markers of thromboembolic risk.

## Introduction

Venous thromboembolism (VTE) includes pulmonary embolism and deep vein thrombosis. It results from changes in blood composition, blood flow and/or changes in the vessel wall. VTE is a common disease with an annual incidence of approximately 1 in 1,000 individuals in developed countries. It involves genetic and environmental risk factors and their interactions [1].

Previous studies [2–4] have estimated a heritability of approximately 60% for the risk of VTE. Well-established genetic risk factors for VTE are unfavourable genotypes at the *SERPINC1*, *PROC*, *PROS1*, *F2*, *FGG*, *F5* and *ABO* loci [5]. The known genetic variants involved in the risk of VTE explain only a small proportion of the genetic variance (heritability). Factor V Leiden (rs6025) and *F2* G20210A (rs1799963) mutations are the most commonly used markers in clinical practice. The variance in the risk of VTE explained by these genetic variations is approximately 7% [6]. Recently, some genetic scores have been designed to predict the occurrence of VTE [6,7]. Soria *et al*. (2014) explained 15% of the variance in the risk of VTE and included 12 variants located in *F2*, *F5*, *ABO*, *F12*, *F13*, *SERPINA10* and *SERPINC1*. Some of these variants were rare variants.

Currently, both low-frequency and rare variants are probable sources of the unexplained heritability in complex traits [8]. Targeted sequencing in individuals selected from the tails of the normal distribution of quantitative phenotypes can be used to identify variants over the full minor allele frequency (MAF) spectrum. These individuals are more likely to carry alleles that cause loss or gain of function [8,9]. Of note, intermediate phenotypes (those correlated with disease) are statistically more powerful and closer to the action of genes than the liability of diseases [10]. Haemostasis parameters have been used to determine the underlying genetic component of the risk of VTE [11,12]. Specifically, plasma coagulation Factor XI (FXI) levels were considered as an intermediate quantitative phenotype in the Spanish families included in the Genetic Analysis of Idiopathic Thrombophilia 1 (GAIT-1) Project to identify new genetic risk factors that contribute to thrombotic disease [2,13]. Interestingly, FXI is a zymogen of a serine protease that participates in the intrinsic coagulation pathway. This glycoprotein has been described [14] as a potential target for a new strategy for VTE treatment. Plasma FXI levels have a heritability of about 45% and a significant genetic correlation with the risk of VTE [2,13]. The genome-wide association studies (GWAS) of plasma FXI levels performed in the GAIT-1 Project showed significant associations with variants in the *KNG1* and *F11* loci. Those results were replicated in a population-based Swedish cohort [15]. The *KNG1* encodes for a high molecular weight kininogen (HMWK). This gene is located on Chromosome 3q27.3 and is 25,581 bp in length. *F11* is the structural gene of coagulation FXI protein and is located on Chromosome 4q35.2 with a length of 23,718 bp (UCSC, Feb. 2009 GRCh37/hg19 release (http://genome.ucsc.edu/) [16]). Also, there is an association of *KNG1* with activated partial thromboplastin time (aPTT) [17,18] and the risk of VTE [19]. The *F11* locus has been associated with aPTT [18] and FXI levels [20,21]. In addition, there is an association of *F11* with the risk of VTE [20,21] that is explained at least in part by an association with FXI levels after pairwise linkage disequilibrium (LD).

We designed a targeted gene Next Generation Sequencing (NGS) strategy to dissect the genetic variability of *KNG1* and *F11* loci. We studied 110 genetically unrelated individuals from the GAIT-2 Project and we identify the genetic variants that might contribute to the variation of plasma FXI levels.

## Material and methods

### Study subjects

The GAIT-2 Project included 935 individuals in 35 extended Spanish families. These families were selected through a proband with idiopathic thrombophilia. A detailed description of the recruitment and the criteria used for inclusion have been given previously [4,22]. Our study was performed according to the Declaration of Helsinki and adult subjects gave written informed consent for themselves and for their minor children. All procedures were evaluated and approved by the Institutional Review Board of the Hospital de la Santa Creu i Sant Pau, Barcelona, Spain.

A total of 110 individuals genetically unrelated from the GAIT-2 Project were chosen to be sequenced using NGS. A subset sample of 40 unrelated individuals was selected as a discovery sample. Among these 40 individuals, there were 20 with low plasma FXI levels (36–80%) and 20 with high plasma FXI levels (158–250%) compared to the normal range in the clinical diagnosis (55–185%). There were 17 males and 23 females. This discovery sample provided more than 98% probability of detecting variants with a MAF greater than or equal to 0.05 [9]. Thus, variants identified by the NGS in the discovery sample were denoted as “variants of interest” for subsequent association analyses and for putative pathogenic mutation screening. Importantly, the genotypes of the “variants of interest” of individuals from the whole sample of 110 individuals were used for association analyses to increase the statistical power to identify statistically significantly genetic variants.

### Blood collection and phenotype determinations

Blood was collected by venipuncture following a 12-hour fast. Samples were collected in an anticoagulant consisting of 1/10 volume containing 0.129 mol/L sodium citrate. None of the participants was using oral anticoagulants or heparins at the time of blood collection. Platelet-poor plasma was obtained by centrifugation at 2,000 *g* for 20 minutes at room temperature (22±2°C) and used for the phenotype determinations that required fresh samples. The remaining plasma was stored at −80°C. FXI activity levels were assayed with deficient plasma (Diagnostica Stago, Asnières, France). DNA was extracted from whole blood using a standard salting out procedure [23].

### PCR primer design

One short PCR and 9 Long Range (LR) PCRs were carried out to amplify 60,052 bp of genomic DNA encompassing the *KNG1* (GRCh37/hg19 chr3:186,435,098–186,460,678) and *F11* (GRCh37/hg19 chr4:187,187,118–187,210,835) loci. All the PCR amplicons were overlapped within each gene region including all exons, introns, 5’-UTR, 3’UTR and approximately 1,500 bp of the promoter region. Primer sequences, primer positions and PCR amplicon sizes in *KNG1* and *F11* are shown in S1 Table.

The PCR amplicons were tested for target specificity by Sanger sequencing of both strands as described below.

### PCR amplification and normalization

Short PCR was performed with the FastStart High Fidelity PCR System, dNTPack (Roche Diagnostics, Mannheim, Germany). LR-PCR amplifications were performed using the SequalPrep Long PCR Kit with dNTPs (Invitrogen, Thermo Fisher Scientific Inc., MA, USA). PCR master mix conditions for Short and LR-PCR amplifications are described in S2 Table and thermocycling conditions used to obtain the optimum short and LR-PCR amplifications are shown in S3 and S4 Tables.

Short and LR-PCR amplicons of the 110 individuals were separated on 0.7% agarose gel electrophoresis and visualized by SYBR safe (Invitrogen, Thermo Fisher Scientific Inc.) staining. Also, all of the PCR amplicons were quantified using the fluorometer Qubit (Invitrogen, Thermo Fisher Scientific Inc.). A normalized pool of the 10 PCR amplicons was obtained for each individual by combining equimolar amounts. The fluorometer Qubit was used to adjust the 110 PCR pools at 0.2 ng/μl for library preparation.

### Library preparation, sequencing and data analysis

The sequencing libraries were prepared from PCR pools using the Nextera XT DNA Sample Preparation kit (Illumina, San Diego, CA, USA) with double indexing, according to the manufacturer’s protocol. Paired-end sequencing was used to improve the mapping quality [24]. We obtained 110 paired-end libraries that were pooled and simultaneously run on an Illumina Miseq sequencing system (Illumina) by the Miseq sequencing reagent kit v2 of 300 cycles (2x150 bp paired end) (Illumina).

Indexed sequences were de-multiplexed and analyzed individually. Paired sequence files in fastq format were analysed with CLC Genomic Workbench version 6.5 software (CLC Bio—Qiagen, Aarhus, Denmark). The raw data were trimmed with length (minimum 25 bp; maximum 500 bp), ambiguous nucleotide (maximum 2) and quality score (0.05) filters. This software aligns the trimmed reads against a human genome reference (hg19). The read mapping was performed with specific parameter setting (mismatch count, 2; indel count, 3; length fraction, 0.7; similarity fraction, 0.9). Parameters were used for quality-based variant detection (minimum coverage, 30x; minimum variant frequency, 25%). Results in variant call format (VCF) file were used as input for the Illumina VariantStudio Data Analysis version 2.1 Software (Illumina) to annotate the genetic variants.

The amino acid numbering and the nomenclature used to describe the sequence variations followed the international recommendations of the Human Genome Variation Society (HGVS; http://www.HGVS.org).

### Association analysis for common and low-frequency variants

We treated our data (FXI levels) as normally distributed over the 110 samples. The normality of the distribution was tested using the Shapiro-Wilk normality test (W = 0.97623; p-value = 0.052) First, MAFs in the 110 individuals were calculated using the PLINK package version 1.07 [25]. For this association analysis, the common variants were defined as genetic variants with MAF ≥10%. Thus, low-frequency variants were defined as genetic variants with MAF <10% [26]. Then, LD based variant pruning was applied for each variant group (common and low-frequency) using the PLINK package to find informative variants. For this, we used the variance inflation factor (VIF) criterion to check for multi-collinearity of variants and recursively remove variants within a sliding window. The VIF was calculated as 1/(1-R^2^) where R^2^ is the multiple correlation coefficient for a variant regressed on all other variants simultaneously at each step. This method considers the correlation between variants and between linear combinations of them. We used a variant windows size of 30, a number of variants to shift the windows at each step equal to 3 and the VIF threshold equal to 2 (i.e. implies R^2^ of 0.5). This allowed variants greater than this VIF to be removed. Association with plasma FXI levels was performed by using two different approaches: (a) a single linear association for common variants using PLINK package, and, (b) a collapsing method based on sliding window for low-frequency variants using the SNP-set (Sequence) Kernel Association Test (SKAT version 1.0.9) available in R [27]. A single variant test analysis is the standard approach to testing for association between genetic variants. However, this approach is less powerful for low-frequency variants. Thus, we analyze common and low-frequency variants separately in order to guarantee the robustness of the results. In the collapsing method, the sets of variants were obtained by shifting one low-frequency variant to the right within a sliding 2-kb window. SKAT aggregates individual score test statistics of each variant and computes variant-set level p-values, while adjusting for covariates. This allowed different variants to have different directions and magnitudes, including no effects [28]. Both methods were adjusted by age and gender. The association of common variants with plasma FXI levels was adjusted for multiple testing by controlling for family-wise error rate (FWER). Multiple testing included Bonferroni single-step correction, Holm step-down correction, Sidak single-step correction, Sidak step-down correction, Benjamini& Hochberg false discovery rate (FDR) control and Benjamini&Yekutieli FDR control. The FWER in low-frequency variants was controlled using a resampling method. We applied a permutation strategy using 1,000 permutations to determine the empirical p-value for denoting a particular variant-plasma FXI level association as statistically significant. Permutation shuffled the FXI values by maintaining the correlation structure among variants. Finally, we used the clump function in PLINK to identify independent signals among common variants.

### Putative pathogenic mutations screening

The following criteria were used to identify putative pathogenic mutations in the data of genetic variation in the discovery sample: a) whether the variant was rare (allele frequency <1% in 1000 Genomes April 2012 version 3 [29] and NHLBI Exome Variant Server June 2013 ESP6500SI-V2), b) location referring to Variant Effect Predictor version 2.8 data base (intronic mutations located >30 bp into flanking intronic regions of each exon were rejected), and c) MAF in our sample of 110 unrelated individuals <5%, for variants not annotated in 1000 Genomes April 2012 version 3 [29].

### Sanger sequencing validations

Putative pathogenic mutations were validated by conventional Sanger sequencing. Briefly, enzymatic purification was performed using ExoSAP-IT treatment (USB Corporation, Cleveland, OH, USA) and Sanger sequencing of both strands was carried out using the BigDye Terminator version 3.1 Cycle Sequencing Kit (Applied Biosystems, Thermo Fisher Scientific Inc., MA, USA). The ABI Prism 3130 Genetic Analyzer (Applied Biosystems, Thermo Fisher Scientific Inc.) was used for capillary electrophoresis. The sequences were mapped against *KNG1* (GRCh37/hg19 chr3:186,435,098–186,460,678; NM_001102416.2) and *F11* (GRCh37/hg19 chr4:187,187,118–187,210,835; NM_000128.3) loci using CLC Genomic Workbench version 6.5 software. We performed also a co-segregation analysis between validated putative pathogenic mutations and plasma FXI levels in available family members by Sanger sequencing.

### *In Silico* prediction analysis

Functional effects of putative pathogenic mutations that co-segregated with plasma FXI levels were evaluated using the *in silico* prediction software Alamut Visual version 2.6.1 (Interactive Biosoftware, Rouen, France). Potential splicing alterations in intron variants included SpliceSiteFinder, MaxEntScan, NNSplice, GeneSplicer and Human Splicing Finder algorithms. The threshold employed was a variation between the native and the mutation score of more than 10% in at least two different algorithms [30]. Pathogenic impact of the missense variants was analysed using the programs SIFT, PolyPhen-2, Align GVGD and Mutation Taster. Conservation phyloP scores were obtained also. Interpretation of predictive structural effects of the missense mutations was evaluated by using the Project HOPE software [31].

### Replication subjects and genotyping

A total of 250 unrelated patients who had suffered thrombosis from a case-control study of Spanish population samples [32] were designated as independent replication subjects. They were used to genotype putative pathogenic mutations from the discovery sample that might be involved in the risk of VTE. Genotyping was performed using TaqMan technology (Applied Biosystems, Thermo Fisher Scientific Inc.) and run in an ABI 7500 instrument (Applied Biosystems, Thermo Fisher Scientific Inc.).

## Results

### NGS statistics

We amplified 60,052 bp of genomic DNA from 110 individuals of the GAIT-2 Project to study the candidate genes *KNG1* and *F11*. Libraries were prepared and pooled for NGS.

The length of the *KNG1* target region was 29,535 nucleotides. The number of reads that cover this region was 7,746,180 and the percentage of the mapped positions with a depth of coverage above 30x was 98%. Further, the median coverage of the gene per individual was 242x. The length of *F11* region was 25,358 nucleotides, the number of reads covering this region was 3,504,328 and the 99% of the positions had coverage above 30x. The median coverage per individual in the *F11* target region was 131x.

A subset of 40 individuals was selected as a discovery sample. We identified a total of 762 unique biallelic variants from this discovery sample (S5 Table). We identified 504 genetic variants in the *KNG1* locus and 258 genetic variants in the *F11* locus. The percentage of indels in the *KNG1* locus was 7.9% (n = 40) and 8.1% (n = 21) in the *F11* locus. The percentage of exonic variants in the *KNG1* locus was 2.2% (n = 11) and 6.2% (n = 16) in the *F11* locus. We found 30.2% (n = 152) of the variants in the *KNG1* locus that had an allele frequency >1% from all populations of 1000 Genomes data (April 2012 version 3) and from four populations of 1000 Genomes (American, East Asian, African and European). The 20.5% (n = 53) of the variants in the *F11* locus had an allele frequency >1% from all populations of 1000 Genomes data (April 2012 version 3) and from four populations of 1000 Genomes (American, East Asian, African and European). Moreover, the 57.1% (n = 288) of the variants in the *KNG1* locus were not reported in the dbSNP version 137 and the 61.6% (n = 159) of the variants in the *F11* locus were not reported in the dbSNP version 137. The majority of these variants that were not reported in dbSNP version 137 were found within the introns (only 2 out of the 288 variants in the *KNG1* locus were located in the promoter region).

### Association analyses

The genotypes of the 110 unrelated individuals for the 762 genetic variants in the discovery sample were used for association analyses to increase the power to detect significant variant-plasma FXI level associations. Of these 762 variants, 41.47% were common (MAF in our population of 110 individuals ≥10%) and the 58.53% were of low frequency (MAF in our population of 110 individuals <10%). After LD based pruning, 125 common variants and 275 low-frequency variants remained.

**Fig 1:**
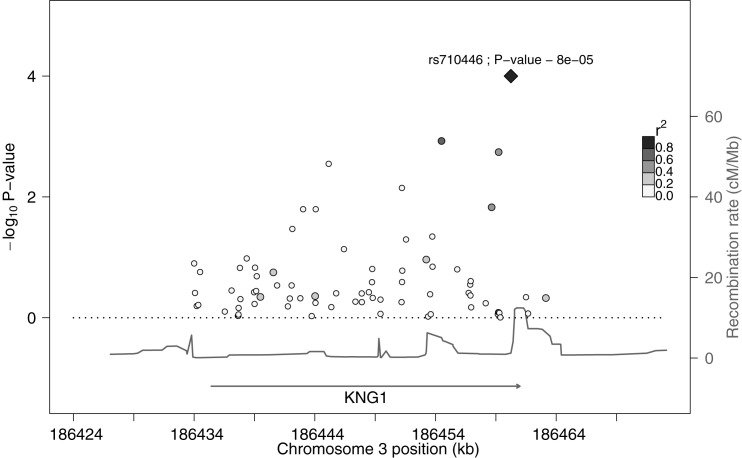
Plot of the association in the *KNG1* locus with plasma FXI levels. Markers represented common variants organized by genomic position. The diamond-shaped marker represented the top SNP (rs710446) which was still statistically significantly associated after adjustment for multiple testing. The left axis shows the statistical significance of the variant-plasma FXI level variation association expressed as -log_10_ of the p-values and colour intensities show the level of linkage disequilibrium between all variants and the top SNP. The recombination rate in the HapMap II sample [33] for this region is measured on the right axis.

**Fig 2:**
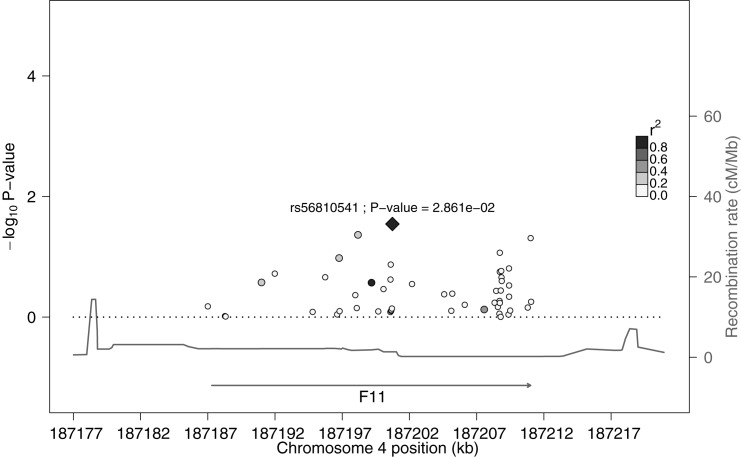
Plot of the association between variants within the *F11* locus with plasma FXI levels. Markers represented common variants organized by genomic position. Tthe diamond-shaped marker represented the top SNP (rs56810541) with the lowest variant-plasma FXI level association p-value. The left axis shows the statistical significance expressed as -log_10_ of the p-values and colour intensities show the level of linkage disequilibrium between all variants and the top SNP. The recombination rate in the HapMap II sample [33] for this region is measured on the right axis.

Using single linear association, the common genetic variant rs710446 was significantly associated with plasma FXI levels after correction for multiple testing. This variant was located in the *KNG1* region. Also, we identified 12 common variants, in addition to rs710446, that showed nominally statistically significant association with plasma FXI levels before correction for multiple testing (p-value <0.05) (Table 1). Of all nominally significantly associated 13 common variants, 10 common variants were located at the *KNG1* locus and 3 common variants were located at the *F11* locus. In detail, 9 out of the 10 common variants in the *KNG1* locus were located in intronic regions and 1 common variant (top SNP in *KNG1* region rs710446) was located within exon 10. In the *F11* region, 2 common variants were located in intronic regions and one variant was located downstream of the locus. The intronic variant rs56810541 was the top SNP in this region. Figs 1 and 2 show plots of the association of common variants with plasma FXI levels. We found 11 independent signals after clumping (rs5030062 and rs3856930 are grouped with the top SNP).

**Table 1:** Significant associations of common genetic variants in *KNG1* (NM_001102416.2) and *F11* (NM_000128.3) with plasma FXI levels.

Gene	Genome Location	Nucleotide Change	dbSNP v137	Tested Allele^1^	MAF (%)^*2*^	β	P-value	Bonferroni-adjusted p-value
*KNG1*	chr3:186,441,823	c.392-1054A>G	rs1656915	A	26.64	-10.9	0.034	1.000
*KNG1*	chr3:186,442,707	c.392-170T>C	rs1648711	T	50.00	-12.1	0.016	1.000
*KNG1*	chr3:186,443,756	c.564+707C>G	rs266724	C	50.00	-12.1	0.016	1.000
*KNG1*	chr3:186,444,831	c.565-195T>G	rs62294376	G	38.32	16.5	0.003	0.355
*KNG1*	chr3:186,450,895	c.930+432T>C	rs1656926	T	29.91	-16.8	0.007	0.887
*KNG1*	chr3:186,453,418	c.930+2955A>T	rs4686800	A	33.18	-8.4	0.045	1.000
*KNG1*	chr3:186,454,180	c.931-2708A>C	rs5030062	C	38.79	15.7	0.001	0.148
*KNG1*	chr3:186,458,322	c.1126-989C>T	rs3856930	T	35.05	12.2	0.015	1.000
*KNG1*	chr3:186,458,910	c.1126-401G>A	rs5030081	A	14.95	23.2	0.002	0.227
*KNG1*	chr3:186,459,927	c.1742T>C	rs710446	C	47.20	19.1	0.00008	0.010
*F11*	chr4:187,197,994	c.755+450C>T	rs4253416	T	46.73	11.5	0.043	1.000
*F11*	chr4:187,200,550	c.756-616A>T	rs56810541	T	36.92	11.6	0.029	1.000
*F11*	chr4:187,210,837	c.*1069delT	rs67843441	A	22.47	10.7	0.049	1.000

^1^ The tested allele is the minor allele by default in our population of 110 individuals.

^2^ The minor allele frequency from our population of 110 individuals, using PLINK package (version 1.07)[25].

**Fig 3:**
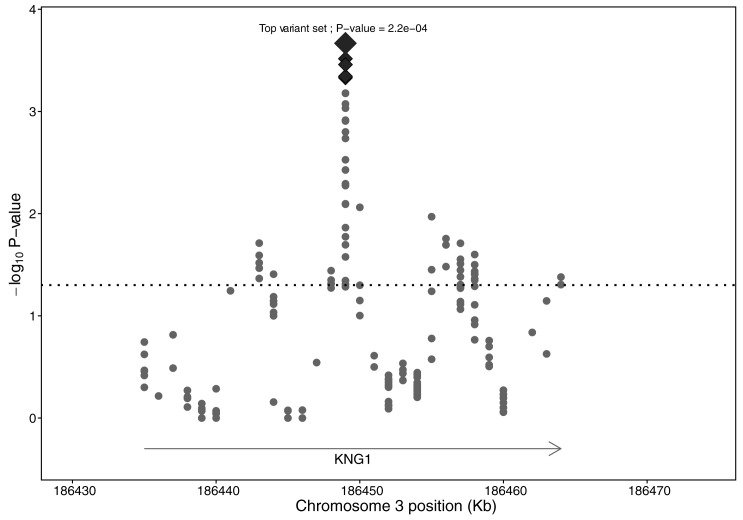
Plot of the collapsing method association in the *KNG1* locus with plasma FXI levels. Markers represented the mean position of low-frequency variant sets. All of the markers located above the dotted horizontal line obtained a p-value <0.05 after the collapsing method association. Diamond-shaped markers represented the five significant low-frequency variant sets with controlling FWER = 0.05. The biggest diamond-shaped marker is the top low-frequency variant set.

**Fig 4:**
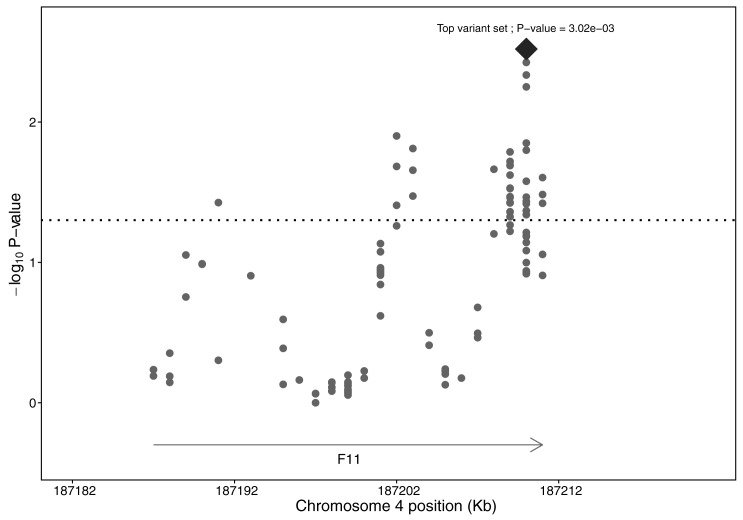
Plot of the collapsing method association in the *F11* locus. Markers represented the mean position of low-frequency variant sets. The diamond-shaped marker represented the top low-frequency variant set and markers located above the dotted horizontal line are the most significant low-frequency variant sets (p-value <0.05). None of the low-frequency variant sets were significantly associated after controlling FWER = 0.05.

Using the collapsing method, 5 low-frequency variant sets were significantly associated with plasma FXI levels after 1,000 permutation analyses, which was used to control the family-wise error rate (FWER = 0.05). Specifically, the 5 low-frequency variant sets were chr3:186,448,468–186,450,468 (p-value = 0.0002), chr3:186,448,470–186,450,470 (p-value = 0.0003), chr3:186,448,478–186,450,478 (p-value = 0.0005), chr3:186,448,482–186,450,482 (p-value = 0.0005) and chr3:186,448,484–186,450,484 (p-value = 0.0004) and included the following genetic variants (NM_001102416.2): c.673-866A>T, c.673-864A>T, c.673-862A>T, c.673-860delT, c.673-856T>C, c.673-852T>C, c.673-850T>C, c.673-842T>C, c.673-838T>C, c.673-806T>C, c.673-406T>C, c.673-136T>C, c.673-67A>G, c.758-91A>C and c.758-12T>C. Also, we identified an additional 89 nominally significantly associated low-frequency variant sets (p-value <0.05) before 1,000 permutation analyses. By merging overlapped significant variant sets before and after 1,000 permutation analyses, different regions in *KNG1* and *F11* were related to plasma FXI variability. Briefly, in the *KNG1* locus, a total of 4 regions were targeted. The first region (chr3:186,441,571–186,444,798) encompassed the exon 4 and the surrounding intronic areas. The second region (chr3:186,446,561–186,450,528) included exon 6, exon 7 and the introns around them. Specifically, the 5 low-frequency variant sets significantly associated with plasma FXI levels after 1,000 permutation analyses were located within these second region (chr3:186,446,561–186,450,528) in *KNG1*. The next area (chr3:186,454,294–186,458,652) encompassed the exons 8 and 9 and the surrounding intronic region. Finally, the downstream region of *KNG1* was the last targeted region (186,462,745–186,465,081). In the *F11* locus 3 regions were highlighted. The first one (chr4:187,189,805–187,191,805) emerged 2,000 bp of the intron 2. Another targeted region encompassed the exons 8, 9 and 10 and the surrounding intronic areas (chr4:187,200,524–187,204,276) and the last targeted region included the exons 13, 14 and 15, the 3’UTR, the intronic areas around them and the downstream region (chr4:187,207,354–187,212,318). Plot for collapsing method in *KNG1* locus is represented in Fig 3 and plot for collapsing method in *F11* locus is represented in Fig 4.

### Putative pathogenic mutations

Among the 762 variants, 6 were selected as potentially functional mutations without association analyses (Table 2). By Sanger sequencing, all candidate mutations were validated to verify the segregation with the FXI levels. Thereby, 2 mutations remained as candidate pathogenic mutations: the intronic variant NM_001102416.2: c.758-12T>C and the missense variant NM_000128.3: c.943G>A (p.Glu315Lys).

**Table 2:** Validated putative pathogenic mutations in *KNG1* (NM_001102416.2) and *F11* (NM_000128.3).

Gene	Nucleotide Change	Location	dbSNP v137	MAF^1^(%)	Co-segregation Validation
*KNG1*	c.-1438_-1433del	Upstream	-	-	No
*KNG1*	c.-1294G>T	Upstream	rs2651642	NA	No
*KNG1*	c.758-12T>C	Intron 6	-	-	Yes
*F11*	c.1304+12G>A	Intron 11	rs116667976	0.18	No
*F11*	c.943G>A	Exon 9	rs281875257	NA	Yes
*F11*	c.*566G>C	3’UTR	-	-	No

NA: Not Annotated.

^1^ The variant allele frequency from all populations of 1000 genomes data, April 2012 version 3 (www.1000genomes.org) [29].

The putative pathogenic mutation NM_001102416.2: c.758-12T>C (in intron 6) at the *KNG1* locus was in heterozygous state within 1 subject with high plasma FXI levels (188%). This variant has been listed previously as variant 3:186450279 T / C in the Exome Aggregation Consortium (ExAC) Browser (Cambridge, MA, http://exac.broadinstitute.org) with a MAF of 4.12x10^-5^. The predicted variations at the natural acceptor splice site of exon 7 between the native and the mutation score were -3.7% (SpliceSiteFinder score native-mutated: 88.56–85.25 [0–100], threshold: ≥70), -6.6% (MaxEntScan score native-mutated: 6.28–5.86 [0–16], threshold: ≥0), -13.7% (NNSplice score native-mutated: 0.66–0.57 [0–1], threshold: ≥0.4), -12.2% (GeneSplicer score native-mutated: 7.43–6.52 [0–15], threshold: ≥0) and -1.4% (Human Splicing Finder score native-mutated: 91.23–89.98 [0–100], threshold: ≥65). These results suggested that the intronic variant NM_001102416.2: c.758-12T>C might interfere with the recognition of the natural acceptor splice site. Notably, this variant was identified in an individual with high FXI levels from the GAIT-2 Project, so the new putative pathogenic mutation NM_001102416.2: c.758-12T>C in the *KNG1* was genotyped in the case-control study of thrombosis. However, it was not identified in any of the 250 patients.

The variation NM_000128.3: c.943G>A (p.Glu315Lys) in the exon 9 of *F11* was in heterozygous state in one individual with low plasma FXI levels (36%). The predicted effects were tolerated (SIFT score: 1 [1–0], median: 3.45), possibly damaging (PolyPhen-2 score: 0.830 [0–1], sensitivity: 0.84, specificity: 0.93), likely pathogenic (Align GVGD score: C55 [C0-C65], GV: 0.00, GD: 56.87) and polymorphism (Mutation Taster p-value: 1 [0–1]). It was predicted that this putative pathogenic mutation would have a weakly conserved nucleotide (phyloP: 1.09 [-14.1;6.4]). According to Project HOPE, the native glutamic acid and the lysine residue resulted from the mutation differ in size and charge. The lysine residue has the opposite charge (positive) at this position and thus contacts with other molecules are likely disturbed. In addition, the lysine residue is bigger than the glutamic acid residue and, as it is located on the surface of the protein, the mutation affects interactions with other parts of the protein and with other molecules. The p.Glu315Lys variation was located within a domain named “Apple 4”, which is important for binding other molecules. Thus, the mutation could disturb the interaction between domains and could ultimately affect the normal protein function.

## Discussion

We report that the genetic variability of *KNG1* and *F11* loci influences the variation in plasma FXI levels.

Our approach was based on target sequencing from a set of high-fidelity polymerase amplifications using NGS. We performed whole gene sequencing to examine not only exonic regions but also non-coding regions that might have regulatory functions [9,34,35].

The *KNG1* and *F11* loci were covered by a large number of high quality reads (median coverage per individual of 242x for *KNG1* and 131x for *F11*), as it has been suggested that a sequencing depth between 30-35x is already optimal to determine variants [36]. We detected a total of 762 genetic variants at the *KNG1* and *F11* loci in the 40 discovery sample individuals. Most of these variants (58.7%) had not been annotated previously in the dbSNP version 137, similar to what has been reported for other loci [26,37]. In addition, only the 26.9% of the variants had an allele frequency from 1000 Genomes (April 2012 version 3) >1%. Thus, most of the 762 variants would not have been included in a GWAS panel.

We analyzed common variants for association with plasma FXI levels in the 110 unrelated individuals. Most importantly, the missense variant rs710446 (p.Ile581Thr) in *KNG1* was associated significantly with high FXI levels after correction for multiple testing. Of note, this genetic variant has been already described [15] in association with plasma FXI levels as a top SNP in the *KNG1* region. Also, the rs710446 variant has been associated previously [17–19] with aPTT and the risk of VTE. In addition, we found 12 additional variants in *KNG1* and *F11* loci that showed nominal significant associations with FXI levels. Of them, the rs5030062 variant in the *KNG1* locus has been described previously [15] in association with plasma FXI levels. Our results are consistent with these results and extents them.

In our study, the low-frequency variants were grouped according to their position across regions of 2 kb and we could identify different sets of low-frequency variants in association with FXI levels. They could be exposing independent regions that contribute to the phenotypic variability.

Two studies [38,39] have explored the risk of VTE using NGS targeted gene strategy. They support the hypothesis that low-frequency and rare variants may contribute to the risk of VTE. We evaluated a combination of common and low-frequency variants at two different loci that affect plasma FXI levels. Our results demonstrated the complexity of phenotypic variation in a single trait.

The 2 putative mutations that were associated with low and high FXI levels indicated that these mutations could be risk factors of FXI deficiency or of thrombosis. We evaluated whether the intronic variation NM_001102416.2: c.758-12T>C in *KNG1* might increase the risk of disease using 5 *in silico* bioinformatics programs. All 5 of them showed that the mutation might reduce splicing and 2 of them predicted an alteration of more than 10% at the acceptor site. To our knowledge, this is the first time that this putative pathogenic mutation has been described in association with high FXI levels. Our genotype assay in patients who had suffered thrombosis from a case-control study did not identify this mutation in this study of Spanish case-control population as it might be restricted to this family. The relatively small number of patients included in our replication study limits the confidence of our conclusions and larger studies need to be performed. Interestingly, the *in silico* prediction of the missense variant NM_000128.3: c.943G>A in the *F11* locus was not conclusive. According to previously published association data [40], this mutation in heterozygous state is associated with low plasma FXI levels. Thus, it is possible that the *in silico* predictions could be underestimating its pathogenicity. We think it is important to emphasize that the 2 putative pathogenic mutations that we detected were located within 2 low-frequency variant sets (chr3:186,446,561–186,450,528 in *KNG1* and chr4:187,200,524–187,204,276 in *F11*) that were nominally significantly associated with plasma FXI levels. Interestingly, the intronic variation NM_001102416.2: c.758-12T>C in *KNG1* was one of the variants included in the low-frequency variant sets that were significantly associated with plasma FXI levels after permutation testing.

Most of the genetic variants associated with plasma FXI levels that we have described would not be detected by examining only the coding regions of the genes. Therefore, it is clear that the non-coding regions can contribute to the regulation of complex phenotypes. We applied *in silico* tools to clarify this issue and provide evidence that support the pathogenic role. However, because prediction programs have serious limitations, further analyses are needed to determine the functional characteristics of these putative mutations.

In conclusion, the continuous DNA sequence data reported in our study using the targeted gene NGS strategy represent one of the largest bodies of sequence data on individuals for the *KNG1* and *F11* loci. Based on the large variation that we detected in the *F11* and *KNG1* loci among 110 individuals from the GAIT-2 Project, we suggest strongly that an overall effect of several of these genetic variations modulates plasma FXI levels. Clearly, further studies are warranted. The resulting functional genetic variants should be useful to predict the risk of thromboembolic disease.

## Supporting information

S1 Table Primers used for amplification of *KNG1* and *F11* loci. (XLSX)

S2 Table PCR master mix for short and LR-PCR amplifications. (XLSX)

S3 Table Thermocycling conditions for Short PCR amplifications. (XLSX)

S4 Table Thermocycling conditions for LR-PCR amplifications. (XLSX)

S5 Table All the unique biallelic variants identified in the *KNG1* and *F11* loci from the discovery sample. (XLSX)
